# Experience in implementing continuous arterial spin labeling on a commercial MR scanner

**DOI:** 10.1120/jacmp.v6i1.2068

**Published:** 2005-03-17

**Authors:** Theodore R. Steger, Edward F. Jackson

**Affiliations:** ^1^ Department of Imaging Physics The University of Texas M. D. Anderson Cancer Center Unit 56, 1515 Holcombe Blvd. Houston Texas 77030 U.S.A.

**Keywords:** cerebral perfusion, arterial spin labeling, magnetic resonance imaging

## Abstract

Continuous arterial spin labeling (CASL) is a technique for performing quantitative perfusion measurements without the need for exogenous contrast agent administration. This technique has seen limited use in the clinic due to problems of poor sensitivity and the potential for artifacts. In addition, CASL requires the application of long‐duration radiofrequency pulses and the acquisition of a large number of images, which can cause difficulties when implemented on commercial MR scanners. This work details our experience in implementing CASL on a commercial MR scanner for the measurement of cerebral blood flow, including pitfalls regarding hardware, radiofrequency energy deposition, and practical application in human subjects. Results of studies to determine the optimal acquisition procedures are also presented.

PACS number: 87.61.‐c

## I. INTRODUCTION

Arterial spin labeling (ASL) is a noninvasive method for obtaining quantitative perfusion measurements which employs magnetic labeling of endogenous water.^(1)^ ^,^ ^(2)^ By subtracting images acquired with (label state) and without labeling (control state), a quantitative perfusion image may be obtained without the need for exogenous contrast agent administration. Arterial spin labeling has been demonstrated in several organ systems, but it is particularly useful for determining regional cerebral blood flow (rCBF).^(3)^ Clinical applications include studies of cerebrovascular disease, stroke, tumor, epilepsy, Alzheimer's disease, and brain functional activation studies.^(3)^ A number of variations of ASL have been proposed, primarily differentiated by whether the labeling of spins is performed with long‐duration radio frequency (RF) pulses applied to a thin plane (continuous ASL, CASL) or short‐duration RF pulses applied to thick slabs of tissue (pulsed ASL). Although the RF‐intensive nature of CASL presents a challenge at field strengths above 1.5 T, it has a higher inherent theoretical signal‐to‐noise ratio than pulsed ASL.^(4)^ Continuous labeling is achieved through flow‐induced adiabatic fast passage^(1)^ ^,^ ^(5)^ in which protons flowing perpendicular to the labeling plane are inverted.

Continuous ASL is generally implemented with a control RF pulse created via amplitude modulation of the labeling RF pulse.^(6)^ This allows for multislice CASL acquisition while controlling for magnetization transfer effects. Twenty to 60 label/control image pairs (40 to 120 averages) are typically acquired to allow for signal averaging to boost the perfusion‐induced signal‐to‐noise ratio. A postlabeling delay is generally introduced between the labeling and imaging segments of the CASL sequence to allow for the labeled blood to perfuse the tissue and correct for transit time artifacts.^(7)^ In addition, a separate $T_{1}$ mapping sequence is typically acquired to facilitate rCBF quantification.

Since its introduction in 1992, much research has been done on ASL techniques, but ASL has seen limited use in the clinic. There are two main limitations preventing its widespread use. First, the theoretical perfusion‐induced signal change for ASL at 1.5 T is just 1% for gray matter and 0.4% for white matter.^(3)^ This low sensitivity makes ASL susceptible to signal changes arising from phenomena other than perfusion. These phenomena include arterial signal artifacts, magnetization transfer artifacts, and the influence of noise in the input images. Second, the need for long‐duration RF pulses and acquisition of many images makes implementation on many commercial scanners difficult. Some groups have attempted to use a separate RF coil to apply the labeling RF pulses, but the need for additional hardware and the ability to decouple the labeling and imaging coils has limited the practical implementation of this two‐coil technique.^(8)^ ^,^ ^(9)^

In this work we present our experience in implementing CASL on a commercial MR scanner. We present several pitfalls regarding hardware concerns, RF deposition issues, and the practical aspects of implementing CASL on human subjects at 1.5 T. We also report the results of our studies performed to optimize the CASL acquisition parameters. In addition, we present our initial experience with CASL on a commercial 3 T scanner.

## II. MATERIALS AND METHODS

A CASL sequence was written using an amplitude modulated control for use with 1.5 T General Electric (GE) Signa scanners (Milwaukee, WI). The sequence used a train of 76 ms RF pulses separated by a gap to accommodate the duty cycle limits of the RF amplifiers. This resulted in pseudocontinuous labeling of arterial protons with a slightly reduced inversion efficiency relative to true continuous labeling. A labeling RF amplitude of 40 mG and labeling gradient of 0.5G/cm were selected based on numerical integration of the Bloch equations. In vivo studies were performed to obtain optimal values for the modulation frequency, postlabeling delay, offset between the labeling plane and imaging slab, and the number of label/control pairs. For GE EPIC software releases prior to 11.0, the number of images per series was limited to 512. This forced a trade‐off between the number of slices and the number of label/control pairs (and hence imaging time). Human volunteers aged 25 to 31 years were screened for MR contraindications and provided informed consent prior to scanning.

The result of the optimization studies was a CASL sequence with 1.8‐s tagging duration, 125‐Hz amplitude modulated control, and 1‐s postlabeling delay. A field of view of 24 cm, matrix size of 64×64, ±62.5 kHz receiver bandwidth, an echo time (TE) of 19.7 ms, and a repetition time (TR) of 3.9 s were used with a gradient recalled echo‐echo planar imaging sequence with 90° flip angle. A slice thickness of 8 mm with an interslice gap of 2 mm was used. The $T_{1}$ mapping was performed using an eight‐point inversion recovery‐echo planar imaging sequence with inversion times of 50 ms, 100 ms, 200 ms, 400 ms, 700 ms, 1000 ms, 2000 ms, and 4000 ms. Two‐dimensional time‐of‐flight angiography was also acquired to help in determining the appropriate labeling plane location.

During the course of the studies, an upgrade to several scanners resulted in the installation of solid‐state RF amplifiers. The previous amplifiers, Erbtec Engineering, Inc. (Boulder, CO) wave tube RF amplifiers, were replaced by Analogic Corp. (Peabody, MA) solid‐state RF amplifiers. We were able to obtain an 83.5% RF duty cycle with the tube amplifiers, but just a 61.2% RF duty cycle with the solid‐state amplifiers. This will be discussed further below.

## III. RESULTS

The CASL sequence was implemented on GE scanners operating at several different EPIC software release levels (8.4, 9.1, G3). Preliminary results indicated that the sequence produced reasonable perfusion‐weighted contrast, although the image quality of the rCBF maps was in need of improvement. Unfortunately, there is no true “gold standard” for human rCBF quantification. In fact, the only consensus in the ASL literature is a relatively consistent value of 2.6 for the gray matter to white matter rCBF ratio.^(10)^ The ratio calculated from the initial results was near 2.6, although further optimization was deemed necessary to increase the image quality.

**Figure 1:**
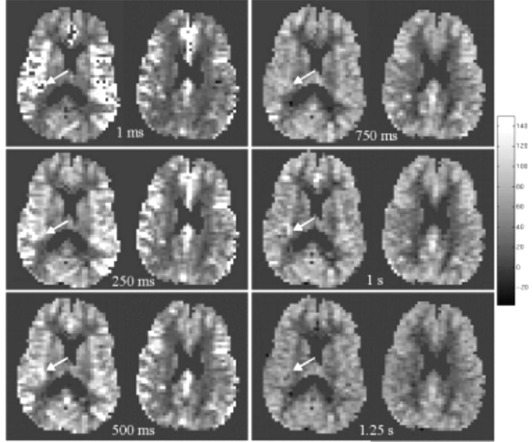
Optimization of postlabeling delay. The inferior‐most rCBF maps from six‐slice CASL acquisitions with postlabeling delays of 1 ms, 250 ms, 500 ms, 750 ms, 1 s, and 1.25 s. The bright arterial signal artifact is largely eliminated for postlabeling delays of 750 ms and greater as indicated by the arrows. The distance from labeling plane to the center of the imaging region for these acquisitions was 80 mm. rCBF is reported in units of mL/100 g/min.

**Figure 2:**
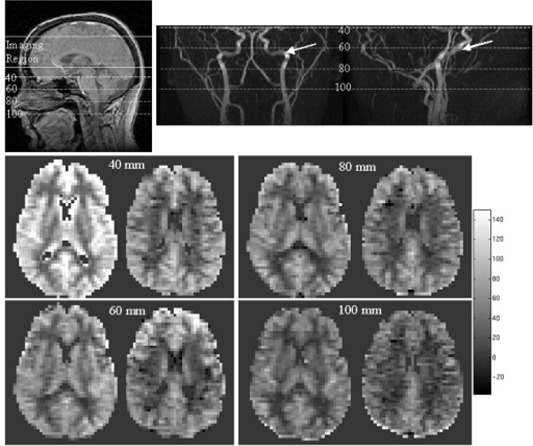
Optimization of the labeling plane to center of imaging region distance. The two most inferior rCBF maps from six‐slice CASL acquisitions with labeling plane to the center of the imaging region distances of 40 mm, 60 mm, 80 mm, and 100 mm. The postlabeling delay for this series of images was 1 s. Also shown is the sagittal localizer image, with the position of the imaging region and each labeling plane noted. Sagittal and coronal 2D time‐of‐flight angiography images are also shown with the location of the labeling planes. The arrows point to the region where the vasculature is not oriented perpendicular to the labeling plane. rCBF is reported in units of mL/100 g/min.

**Figure 3:**
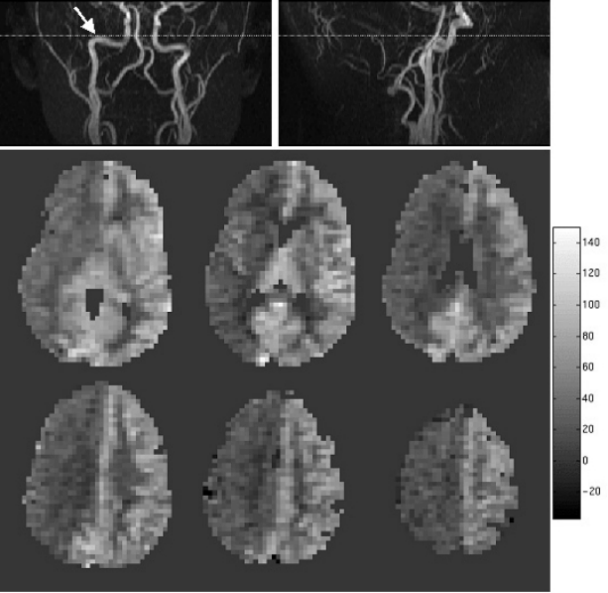
Demonstration of the effect of poor labeling plane location selection. All six rCBF maps from a six‐slice CASL acquisition are shown for a subject positioned with her head slightly askew. Also shown are the sagittal and coronal 2D time‐of‐flight angiography images with the labeling plane shown by the horizontal line. The arrow points to the region where the vasculature is oriented parallel to the labeling plane resulting in greatly reduced calculated rCBF values for the subject's right hemisphere. rCBF values are reported in units of mL/100 g/min.

**Figure 4:**
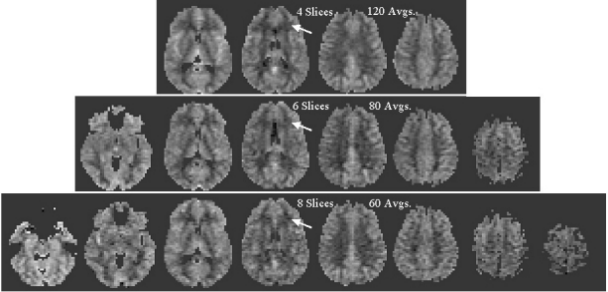
Analysis of the trade‐off between the number of slices and the number of averages. rCBF maps for four‐, six‐, and eight‐slice CASL acquisitions with 120, 80, and 60 averages, respectively. The arrows indicate an area of decreasing image quality with increased number of slices. A postlabeling delay of 1 s and labeling plane to center of imaging region distance of 80 mm were used. rCBF values are given according to the scale of the previous figures.

The results of the optimization studies are shown in Figs. 1 to 4. Figure 1 depicts the rCBF maps for the inferior‐most slice of a six‐slice acquisition at postlabeling delays of 1 ms, 250 ms, 500 ms, 750 ms, 1 s, and 1.25 s. It is apparent that the bright arterial signal artifact is reduced for delays greater than 500 ms for the 80 mm label/image offset used in this study (see arrows). Note that this study was performed on relatively young healthy subjects. To account for possible increased transit time in a patient population, a postlabeling delay of 1 s or greater is recommended.

Figure 2 shows rCBF maps for the two most inferior slices of a six‐slice acquisition for different values of the offset distance between the labeling plane and the center of imaging slab. In addition, the location of the labeling planes is shown on the sagittal localizer images and a 2D time‐of‐flight angiography sequence. Remember that adiabatic fast passage labeling is most efficient when flow is perpendicular to the labeling plane. For the 40‐mm offset distance there is likely perturbation of the most inferior slice by the labeling RF pulses producing an rCBF map with artificially elevated rCBF. The angiographic study reveals that the vasculature is oriented obliquely to the labeling plane for the 60‐mm offset distance. This results in decreased inversion efficiency and hence lowered image quality. For labeling planes located at 80‐mm and 100‐mm offset distances the vasculature is predominantly perpendicular to the labeling plane, and higher‐quality rCBF maps are obtained.

Figure 3 depicts a situation in which the subject was not properly positioned in the scanner. The subject had a slight tilt of the head, causing the subject's right middle cerebral artery to run parallel to the labeling plane while the left middle cerebral artery was perpendicular to the labeling plane. This resulted in an inversion efficiency close to zero for the subject's right middle cerebral artery and produced artificially low rCBF values for the brain regions perfused by this artery. For this reason, it is recommended that an angiography sequence be performed to verify appropriate placement of the labeling plane.

The 512 image per series limit placed an upper bound on the number of averages that could be acquired for a given number of slices. The number of slices (along with slice thickness and slice spacing) defines the anatomic coverage, while the number of averages is related to the scan duration. Figure 4 shows a comparison of rCBF maps acquired with a four‐slice acquisition (120 averages, 7:20 scan time), a six‐slice acquisition (80 averages, 5:10 scan time), and an eight‐slice acquisition (60 averages; 3:42 scan time). The relative merits of the number of averages and number of slices are evident in the figure. The four‐slice acquisition demonstrates excellent image quality but limited anatomic coverage. The eight‐slice acquisition shows reduced image quality (see arrow) due primarily to the reduced signal averaging. Its anatomic coverage is greater, although the most superior and inferior slices are unlikely to be areas of clinical interest. The six‐slice acquisition seems to be a good compromise between the number of signal averages and the anatomic coverage. The 512‐slice limit is not a concern for EPIC releases later than 11.0, so the number of averages on scanners running 11.0 or greater is limited solely by clinically feasible scan times.

## IV. DISCUSSION

In the course of implementing the CASL sequence, several difficulties were encountered. First among them were issues with the RF amplifier duty cycle. For ideal continuous inversion, RF pulses several seconds in duration would be required. For single‐coil CASL on a clinical scanner, RF amplifiers do not allow for such long‐duration pulses. By separating the single pulse into several shorter‐duration pulses separated by a small gap, the duty cycle constraints can be alleviated at the expense of inversion efficiency. For the wave tube RF amplifiers, a 15‐ms gap between each of twenty 76‐ms pulses created a duty cycle of 83.5%. With the solid‐state amplifiers, a 48‐ms gap was required, leading to a long labeling time, reduced duty cycle of 61.2%, and proportionally lowered inversion efficiency. Communication with the vendor revealed no alternative to increase the RF amplifier duty cycle for the solid‐state amplifier. Qualitatively, our results with the solid‐state amplifier were only slightly degraded. Nonetheless, when possible we performed our experiments using the wave tube amplifiers. We point out this issue so that sites attempting to implement CASL may be aware of possible hardware concerns with the RF amplifier type. Two‐coil CASL has lowered RF duty cycle concerns, but the additional hardware required makes it less than ideal for clinical implementation. It is also recommended that the maximum pulse length allowed by the RF amplifier be obtained from the vendor in order to optimize the pseudocontinuous labeling technique.

The second difficulty encountered dealt with placement of the labeling plane. Although it was expected that the morphology of the vasculature would allow for a constant labeling‐to‐imaging plane distance, we found several instances with degraded perfusion‐induced signal change, likely due to decreased labeling efficiency from the oblique orientation of the vasculature relative to the labeling plane. With this in mind, we recommend performing an MR angiography scan to ensure appropriate placement of the labeling plane. A 2D time‐of‐flight sequence covering the labeling region of interest typically required 2 min to 3 min of scan time.

Although this difficulty has been rectified with later software releases, GE scanners running versions before 11.0 are subject to a 512 image per series limit. This limit forced a trade‐off between number of slices and number of averages. We recommend 6 slices and 80 averages, although if the limit were not in place, additional averages and slices would be appropriate, depending on the anatomic region of interest and desired scan time.

A final concern involves RF deposition limits. Our implementation of CASL at 1.5 T produced calculated specific absorption rates (SAR) significantly below the limits imposed by the U.S. Food and Drug Administration. However, we did expect to encounter difficulties with SAR during implementation on a 3 T GE Signa scanner. A preliminary study found that our CASL sequence with 60 averages and twenty 76‐ms RF pulses separated by a 48‐ms gap generated SAR values right at the limit of the RF power monitor. We intend to reduce the magnitude of the labeling RF and labeling gradient from 40 mG and 0.5G/cm to 25 mG and 0.25G/cm, respectively, to reduce the SAR while maintaining approximately the same inversion efficiency.^(11)^ This should allow us more flexibility in terms of the number of allowable averages, which is particularly important for perfusion‐based functional MRI applications.

## V. CONCLUSIONS

In this work we have presented our experience in implementing single‐coil CASL on a commercial scanner. Several vendor‐specific difficulties were encountered, although we expect that others may experience similar difficulties with hardware from other vendors. In addition, optimization of the CASL sequence was demonstrated to illustrate several pitfalls and recommendations for other sites interested in implementing CASL.

## ACKNOWLEDGMENTS

This work was funded in part by the John S. Dunn Foundation. The support of William A. Murphy, Jr., MD, is gratefully acknowledged.
